# Magnetic and Mössbauer Spectroscopy Studies of Zinc-Substituted Cobalt Ferrites Prepared by the Sol-Gel Method

**DOI:** 10.3390/ma11101799

**Published:** 2018-09-21

**Authors:** Qing Lin, Jianmei Xu, Fang Yang, Jinpei Lin, Hu Yang, Yun He

**Affiliations:** 1 College of Medical Informatics, Hainan Medical University, Haikou 571199, China; elinqing@126.com (Q.L.); linqinglab@126.com (J.X.); heyunlab@163.com (F.Y.); 2 Guangxi Key Laboratory of Nuclear Physics and Nuclear Technology, Guangxi Normal University, Guilin 541004, China; 3 College of Physics and Technology, Guangxi Normal University, Guilin 541004, China; yanghu000@126.com; 4 Sate Key Laboratory for Chemistry and Molecular Engineering of Medicinal Resources, Guangxi Normal University, Guilin 541004, China

**Keywords:** Co-Zn-ferrite, sol-gel auto-combustion, Zn substitution, mössbauer, magnetic properties

## Abstract

Zinc ion-substituted cobalt ferrite powders Co_1−*x*_Zn*_x_*Fe_2_O_4_ (*x* = 0–0.7) were prepared by the sol-gel auto-combustion process. The structural properties and magnetic of the samples were investigated with X-ray diffraction (XRD), superconducting quantum interference device, and a Mössbauer spectrometer. The results of XRD showed that the powder of a single cubic phase of ferrites calcined when kept at 800 °C for 3 h. The lattice constant increases with increase in Zn concentration, but average crystallite size does not decrease constantly by increasing the zinc content, which is related to pH value. It was confirmed that the transition from ferrimagnetic to superparamagnetic behaviour depends on increasing zinc concentration by Mössbauer spectra at room temperature. Magnetization at room temperature increases for *x* ≤ 0.3, but decreases for increasing Zn^2+^ ions. The magnetization of Co_0.7_Zn_0.3_Fe_2_O_4_ reached maximum value (83.51 emu/g). The coercivity decreased with Zn^2+^ ions, which were doped on account of the decrease of the anisotropy constant.

## 1. Introduction

Cobalt ferrite is important magnetic material. It is a hard ferromagnetic material, which has high coercivity of 5000 Oe, a high Curie temperature (T_C_) of 520 °C, moderate saturation magnetization of approximately 80 emu/g, a high anisotropy constant of 2.65 × 10^6^–5.1 × 10^6^ J/m^3^, and a high magneto-strictive of −225 ppm [1,2]. Moreover, cobalt ferrite exhibits high electromagnetic performance, a large magneto-optic effect, excellent mechanical hardness, and chemical stability [3]. Cobalt ferrite has been widely used as a high-density recording medium, because it is a well-known hard magnetic material [4]. Furthermore, cobalt ferrite is a promising candidate for medical applications, such as magnetic drug delivery, magnetic resonance imaging (MRI), radio-frequency hyperthermia and medical diagnostics [2], microwave and magneto-optic devices, and high-frequency catalysis and applications [1,3].

The saturation magnetization, electrical resistivity, coercivity, permittivity, and permeability of cobalt ferrite can be modified with partial replacement of non-magnetic zinc cations. The Zn^2+^ ion of non-magnetic substituted cobalt ferrite leads to a decrease in its saturation magnetization, Curie temperature, and coercivity [2]. Direct current (DC) electrical resistivity increases and the dielectric constant of the ferrite Co_1−*x*_Zn*_x_*Fe_2_O_4_ decreases with an increase in Zinc content, but the DC electrical resistivity decreases by increasing the calcination temperature, which ensures the semi-conductor performance of the sample [5]. Literature [6] has reported the permittivity and permeability of Co_0.5_Zn_0.5_Fe_2_O_4_ between 10 MHz to 1.0 GHz, which shows that the material is a potential wave absorber of electromagnetic interference (EMI). Veverka et al. [7] studied ferrite Co_1−*x*_Zn*_x_*Fe_2_O_4+γ_; when *x* = 0.6, they observed a transition temperature of 310–334 K for the paramagnetic state, which suggests that the magnetic fluid hyperthermia can be applied in a self-controlled regime. The maximum magnetoresistance (MR) has been observed for the Zn_0.8_Co_0.2_Fe_2_O_4_ polycrystalline sample [8].

In this paper, ferrite powders Co_1−*x*_Zn*_x_*Fe_2_O_4_ (*x* = 0–0.7) were synthesized with the sol-gel auto-combustion method. The aim was to study variation in structural and magnetic performance of cobalt ferrite powders with partial substitution of non-magnetic zinc cations.

## 2. Experimental

### 2.1. Sample Synthesis

Zinc ion-substituted cobalt ferrite powders Co_1−*x*_Zn*_x_*Fe_2_O_4_ (*x* = 0–0.7) were synthesized with the chemical method of sol-gel auto-combustion. The raw materials were analytical grade Co(NO_3_)_2_·6H_2_O, Zn(NO_3_)_2_·6H_2_O, Fe(NO_3_)_3_·9H_2_O, citric acid (C_6_H_8_O_7_·H_2_O), and ammonia (NH_3_·H_2_O). The molar ratio of citric acid to metal nitrates was 1:1. The citric acid and metal nitrates were respectively added to deionized water. Ammonia was added to adjust the pH value (about 7) of the metal nitrates solution. The solution was put into a thermostat water bath and maintained at 80 °C under continuous stirring to form the dried gel. In the process of stirring, citric acid was dripped continually. The gel was dried in a dry-oven of 120 °C for two hours, being burnt from the self-propagating combustion to become loose powder. The loose powder was ground and calcined at 200 °C, 400 °C, 600 °C, and 800 °C for 3 h.

### 2.2. Characterization

The structure and crystallite sizes were characterized by XRD (D/max-2500V/PC, Rigaku Corporation, Tokyo, Japan) in the 2θ range 20–70° using Cu-K_α_ radiations (λ = 0.15405 nm). The crystallite sizes were calculated with Scherrer’s formula: D = kλ/hcosθ where D, k, h, and θ are the average diameter, the shape factor, the half intensity width of the relevant diffraction peak, and diffraction angle, respectively. The micrographs were obtained by scanning electron microscopy (Nova NanoSEM 430, FEI Corporation, Hillsboro, OR, USA). Saturation magnetization was measured by Quantum Design MPMS series XL-7(Quantum Design Corporation, San Diego, CA, USA). The Mössbauer spectrum was performed by Mössbauer spectroscop (Fast Tec PC-mossII, FAST Corporation, Oberhaching, Bavaria, Germany, in constant acceleration mode. The γ-rays were provided by a ^57^Co source in a rhodium matrix. The hyperfine parameters, magnetic hyperfine field (H_hf_), isomer shift (I.S.), quadrupole shift (Q.S.), relative area (A_0_), and line width (Г) were obtained by the fitted spectra through the Mösswinn 3.0 program, and the calibration was relative to 25 μm thick high purity alpha iron.

## 3. Results and Discussion

### 3.1. X-ray Diffraction Analysis

**Figure 1:**
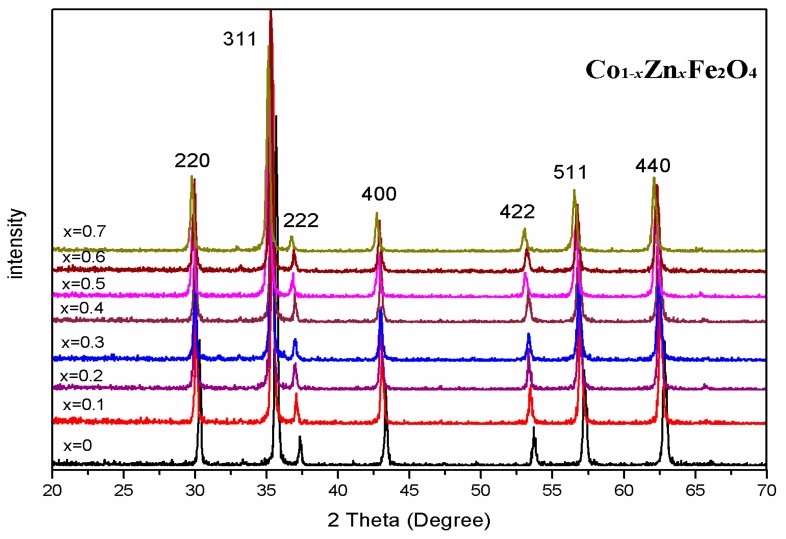
Room-temperature XRD patterns of Co_1−*x*_Zn*_x_*Fe_2_O_4_ calcined at 800 °C.

The X-ray diffraction (XRD) patterns of Co_1−*x*_Zn*_x_*Fe_2_O_4_ (*x* = 0–0.7) ferrites calcined at 800 °C for 3 h are shown in Figure 1. The XRD of the samples exhibited single-phase spinel structure. No impurity peak was detected in these samples. Table 1 shows that the lattice parameter increased by increasing the zinc content. The increase of the lattice parameter is probably due to the radius of the Zn^2+^ ions (0.74 Å) being larger than that of the Co^2+^ ions (0.72 Å) [8,9]. Zn^2+^ ions prefer to enter the tetrahedral A site, while Co^2+^ ions prefer to enter the octahedral B site in the Co_1−*x*_Zn*_x_*Fe_2_O_4_ ferrite [10,11]. On the basis of the earlier studies [12], the spinel structure can be assigned in the synthesized materials, which can be expressed as:

(1) $$({\mathrm{Zn}}_{x}^{2+}{\mathrm{Fe}}_{1-x}^{3+})[{\mathrm{Co}}_{1-x}^{2+}{\mathrm{Fe}}_{1+x}^{3+}]O_{4}^{2-},$$

where the parentheses indicate cations in the tetrahedral A sites, and the cations in the square bracket are in the octahedral B sites. The theoretical lattice parameter (*a_th_*) was estimated using the relation related to the radii of the tetrahedral and octahedral sites (*r_A_*, *r_B_*) [9]:

(2) $$a_{th}=\frac{8}{3\sqrt{3}}\left[\left(r_{A}+R_{0}\right)+\sqrt{3}\left(r_{B}+R_{0}\right)\right],$$

where *R*_0_ is the oxygen radius (*R*_0_ = 1.32 Å) [10,11,12], *r_A_* and *r_B_* are the tetrahedral radii and octahedral radii, respectively. On the basis of the ion distribution model (1), *r_A_* and *r_B_* are calculated:

(3) $$\begin{array}{l} r_{A}=x\;r_{{\mathrm{Zn}}^{2+}}+(1-x)r_{{\mathrm{Fe}}^{3+}}\\ r_{B}=\frac{1}{2}[(1-x)\;r_{{\mathrm{Co}}^{2+}}+(1+x)r_{{\mathrm{Fe}}^{3+}}] \end{array}$$

**Figure 2:**
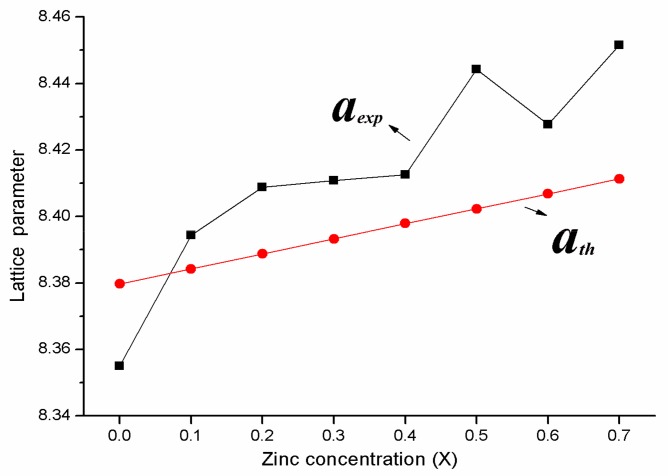
The variation of the theoretical and experimental lattice parameter for Co_1−*x*_Zn*_x_*Fe_2_O_4_ (*x* = 0–0.7).

We estimated the value of *a_th_* using Equations (2) and (3). There are deviations between the theoretical and the experimental lattice parameter, because cobalt ions (Co^2+^) not only occupy the octahedral site (B), but a few cobalt ions (Co^2+^) occupy the tetrahedral site (A) [10]. However, the experimental lattice parameter *a_exp_* and the theoretical lattice parameter *a_th_* all increase with the increasing of Zn^2+^ ions, as shown in the Figure 2. Average crystallite size tends to decrease with the increasing of Zn concentration, for the following reasons [13]: when zinc is introduced into the system, it will liberate more heat and decrease the crystal surface molecular concentration, thereby have a large impact on the grain growth. The preferences of the cations are not fully satisfied, which may be obstructed by grain growth. The higher bond energy of Co^2+^-O^2−^, as compared with Zn^2+^-O^2−^, lead to the particle sizes of the samples decrease with the increasing of zinc content. However, average crystallite size not decrease continuously with an increase in Zn ions, and is related to the pH value. When the pH value is too high, there is a lot of gas created by the ammonia, leading to an increase in porosity and a decrease in average crystallite size [14]. 

The X-ray density was estimated by the following relation [15]:

(4) $$\rho_{x}=\frac{8M}{Na^{3}},$$

where *M* is relative molecular mass, *a* is the lattice parameter, and *N* is the Avogadro’s number. Table 1 shows the X-ray density decreases with a Zn^2+^ concentration for *x* ≤ 0.4. The atomic weight of zinc is greater than the cobalt, so the increase of the lattice parameter leads to the decrease in X-ray density.

**Figure 3:**
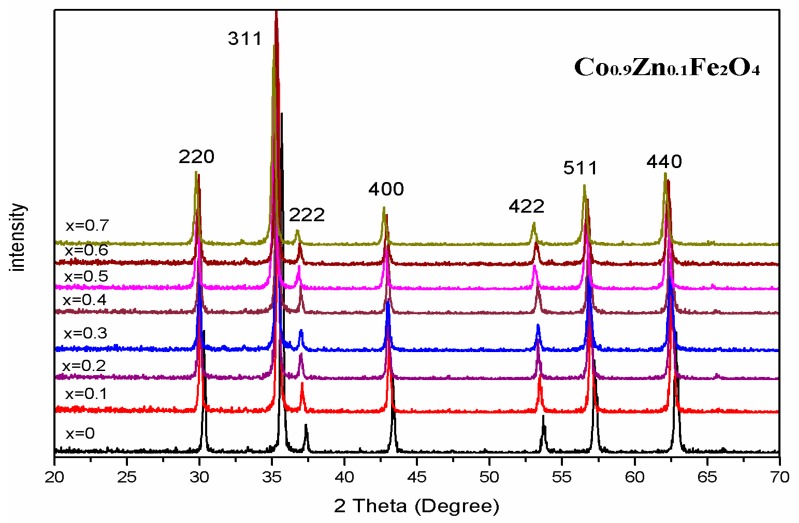
The XRD of Co_0.9_Zn_0.1_Fe_2_O_4_ sintered at different temperatures.

The XRD of Co_0.9_Zn_0.1_Fe_2_O_4_ sintered at different temperatures are shown in Figure 3. All the samples are of the single-phase cubic spinel structure. Table 2 shows the X-ray density of Co_0.9_Zn_0.1_Fe_2_O_4_ sintered at different temperature. The additional phase was not detected. For all the samples, the lattice parameter showed no significant changes. The average crystallite size of Co_0.9_Zn_0.1_Fe_2_O_4_ increases with an increase in the calcining temperature. When the burning temperature is below 600 °C the diffraction peaks of Co_0.9_Zn_0.1_Fe_2_O_4_ in our result were sharper and narrower relative to the diffraction peaks of Co_0.5_Zn_0.5_Fe_2_O_4_ in [11]. It indicates that when we prepared zinc-substituted cobalt ferrite powders with the experimental method of sol-gel auto-combustion, the sample without calcining had a good crystallinity [16]. 

### 3.2. Structures and Grain Sizes

**Figure 4:**
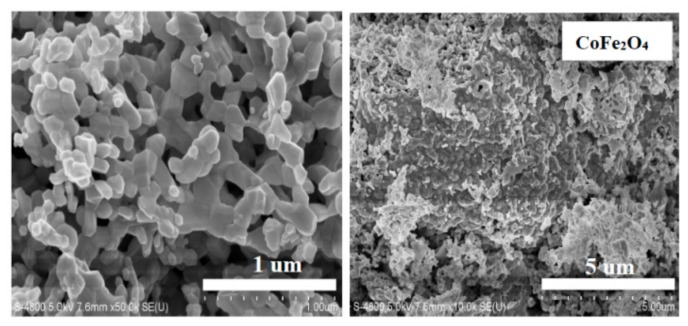
Scanning electron microscopy (SEM) micrographs of CoFe_2_O_4_ annealed at 800 °C.

**Figure 5:**
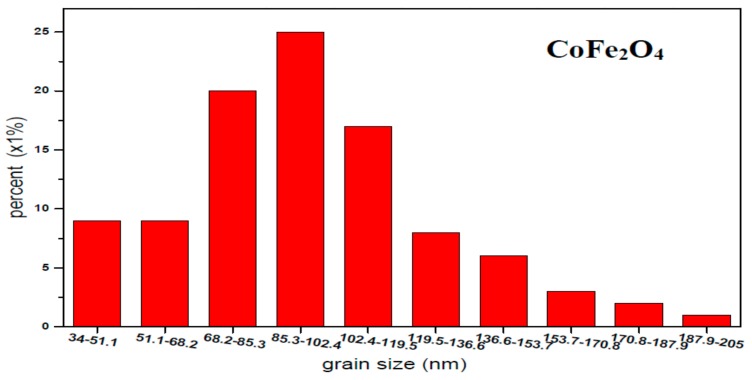
Histogram of grain size distribution of CoFe_2_O_4_ annealed at 800 °C.

The micrographs of CoFe_2_O_4_ annealed at 800 °C from scanning electron microscopy (SEM) are shown in Figure 4. It can be observed that the distribution of grains were almost uniform in size and well crystallized. Figure 5 shows the histogram of the grain size distribution of CoFe_2_O_4_ ferrites. The average grain size of CoFe_2_O_4_ estimated by a statistical method is approximately 96.26, which shows that CoFe_2_O_4_ ferrite powers are nanoparticles.

### 3.3. Mössbauer Spectroscopy

**Figure 6:**
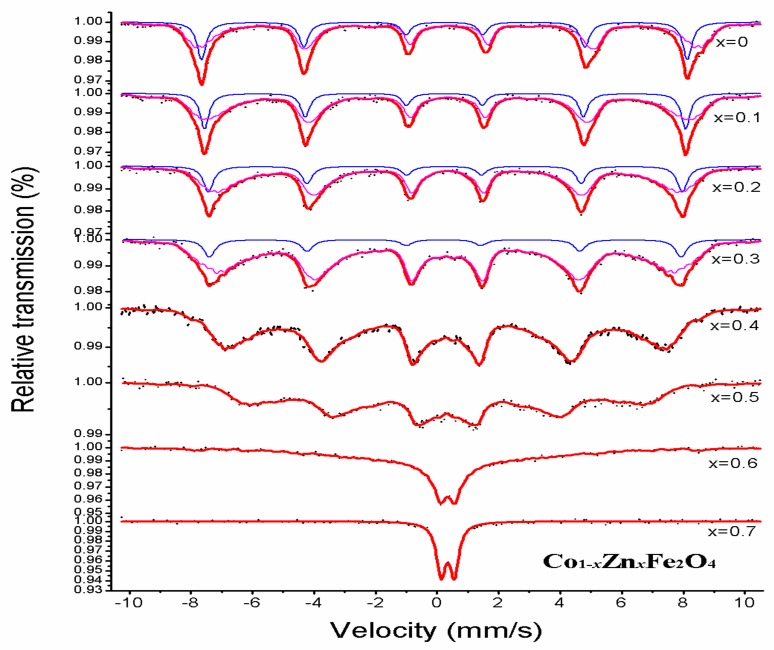
The Mössbauer spectra of Co_1−*x*_Zn*_x_*Fe_2_O_4_ sintered at 800 °C.

The Figure 6 shows the Mössbauer spectra at room temperature for Co_1−*x*_Zn*_x_*Fe_2_O_4_. The hyperfine parameters, isomer shift (IS), magnetic hyperfine field (H_hf_), quadrupole shift (QS), relative area (A_0_), and line width (Г), were obtained by fitted spectra using the Mösswin 3.0 software program [17], and calibration was relative to a 25 μm thick sample of high purity alpha iron. For the Co_1−*x*_Zn*_x_*Fe_2_O_4_ with 0 ≤ *x* ≤ 0.3, the Mössbauer spectra are two sextets of normal Zeeman splits, which is attributable to Fe^3+^ ions at the tetrahedral A site and octahedral B site, indicating the ferrimagnetic properties of the samples. The sextet are assigned to the Fe^3+^ ions of the tetrahedral A site and the octahedral B site, and the octahedral B site isomer shift is larger than that of tetrahedral A site. Maybe it is due to the fact that the bond separation of A site Fe^3+^ ions is smaller than that of the B site Fe^3+^ ions, which have the smaller orbits overlapping Fe^3+^ ions and O^2+^ ions at the octahedral B site, resulting in smaller covalency for Fe^2+^ ions in octahedral B site [16,17,18]. Other studies have shown that the value of Fe^3+^ (S = 1/2, 3/2, 5/2) ions is 0.1–0.5 mm/s, while for Fe^2+^ (S = 2) ions the value is 0.6–1.7 mm/s [19]. As seen in Table 3, the value of iron is Fe^3+^ state in our study.

The magnetic hyperfine field (H) of A and B sites decreases with an increase in non-magnetic zinc substitution, It was also observed that the H_A_ decreases with a larger rate than H_B_ [20,21], because non-magnetic ion Zn^2+^-substituted cobalt ferrite goes to the A site [11]. In all of the samples, the Mössbauer spectra quadrupole shift is close to zero, which indicates that the ferrite is close to cubic symmetry. The A Mössbauer absorption area decreases and the B Mössbauer absorption area increases with increasing zinc concentration, since Zn^2+^ substitutes for cobalt ferrite and occupies the A site, leading to Fe^3+^ from the A site transferring to the B site. When 0.4 ≤ *x* ≤ 0.6, the spectra of Co_1−*x*_Zn*_x_*Fe_2_O_4_ is only the B magnetic sextet—the magnetic sextet of A site vanishes, which indicates the Fe^3+^ ions only occupy the octahedral B site [22]. When *x* = 0.6, the Mössbauer spectrum showed the relaxation effects features and was fitted one single sextet. When *x* = 0.7, the Mössbauer spectrum of the sample consisted only of a central doublet, and it exhibited superparamagnetic character. The central doublet can be due to the nonmagnetic nearest neighbors of Fe^3+^ ions, as the Fe^3+^ ions of magnetic isolation do not take part in long-range magnetic ordering [22,23,24].

**Figure 7:**
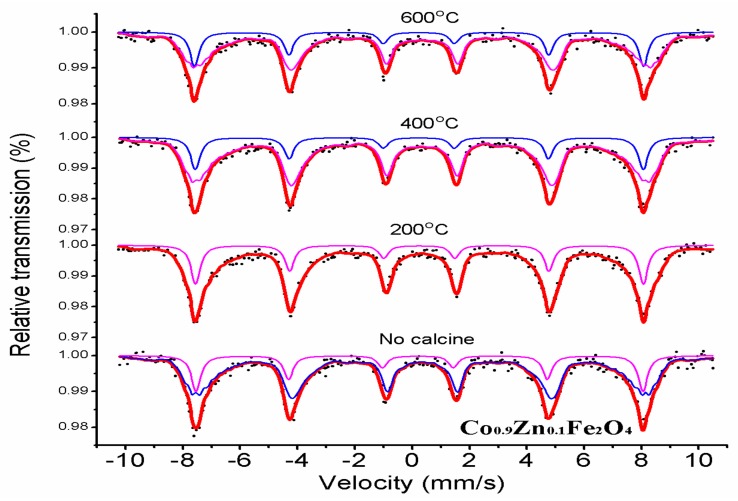
The Mössbauer spectrum of Co_0.9_Zn_0.1_Fe_2_O_4_ calcined at different temperatures.

Figure 7 shows room-temperature Mössbauer spectrums of Co_0.9_Zn_0.1_Fe_2_O_4_ powders calcined at different temperatures. Spectra of all samples sintered at different temperatures were fitted with two sextet sub-patterns. The research results of others [25] show that Mössbauer spectrums of Co_0.9_Zn_0.1_Fe_2_O_4_ calcined at different temperatures display a transition from paramagnetic doublet to ferrimagnetic sextet. Table 4 shows that while the Mössbauer parameters have no significant change for Co_0.9_Zn_0.1_Fe_2_O_4_ calcined at different temperatures, the magnetic hyperfine field increases slightly with increasing annealing temperatures. The X-ray patterns show that Co_0.9_Zn_0.1_Fe_2_O_4_ calcined at different temperatures has a good crystallinity, and that average crystallite size increase with increasing the calcining temperature. Therefore, the magnetic hyperfine field that has changed for the ferrite powders can be attributed to the variation of average crystallite size as a function of sintering temperature [25,26].

### 3.4. Magnetic Analysis

**Figure 8:**
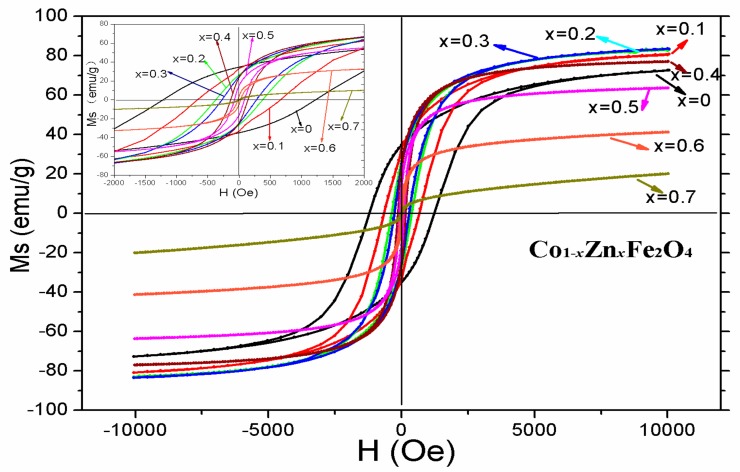
Room temperature hysteresis loops of Co_1−*x*_Zn*_x_*Fe_2_O_4_ calcined at 800 °C.

The room temperature hysteresis loops of Co_1−*x*_Zn*_x_*Fe_2_O_4_ are shown in Figure 8. It is observed from Table 5 that magnetization at 1000 Oe increases initially (up to *x* = 0.3) and then decreases as Zn content *x* increases. 

**Figure 9:**
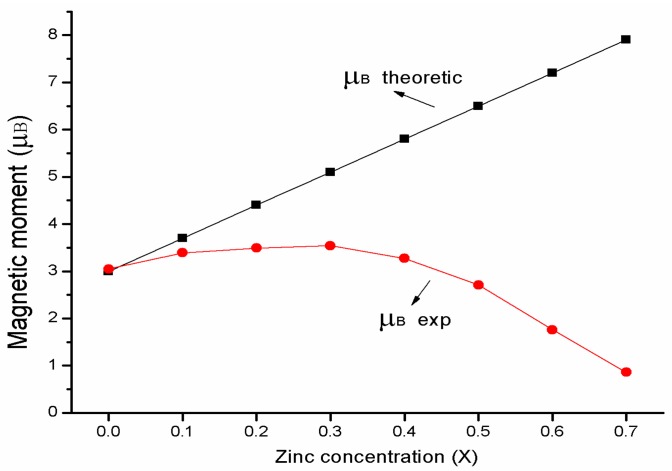
Variation in experimental and theoretical magnetic moment with Zinc content *x*.

When *x* = 0.3, the magnetization maximum value is 83.51 emu/g, the results are almost equal to the literature [11]. The magnetization could be expressed by the following relation [27]:

(5) $$\sigma_{s}=\frac{5585\times n_{B}}{M},$$

where *n_B_* is the magnetic moment and *M* is relative molecular mass. The relative molecular mass of Co_1−*x*_Zn*_x_*Fe_2_O_4_ decreases as Zn content *x* increases. The variation of magnetic moment *n_B_* can be explained with Néel’s theory. The magnetic moment of Fe^3+^, Co^2+^, and Zn^2+^ ions are 5 μ_B_, 3 μ_B_, and 0 μ_B_ [9,11], respectively. According to two sub-lattice models of Néel’s theory, using the cation distribution of $({\mathrm{Zn}}_{x}^{2+}{\mathrm{Fe}}_{1-x}^{3+})[{\mathrm{Co}}_{1-x}^{2+}{\mathrm{Fe}}_{1+x}^{3+}]O_{4}^{2-}$ (1), the magnetic moment *n_B_* is expressed as [9,11,28]:

(6) $$n_{B}=M_{B}-M_{A}=(1+x)\times 5+(1-x)\times 3-(1-x)\times 5=3+7x,$$

where *M_A_* and *M_B_* are the A and B sub-lattice magnetic moments. Figure 9 shows the change in the experimental and theoretical magnetic moment with Zn content *x*. From the Equation (6), the magnetic moment *n_B_* increases with the increasing Zn content, and according to the relation (5) the theoretical magnetization monotonously increases as Zn content *x* increases. The change of the experimental and theoretical magnetization are in a good agreement with each other for *x* ≤ 0.3. When *x* ≥ 0.4, the experimental magnetization decreases as Zn content *x* increases, which can be explained with the Yafet–Kittel (YK) three sub-lattice model [9,29]. It is a reasonable that the sample appears to spin around the arrangement of the magnetic moment on B-sites when the content of nonmagnetic Zn^2+^ substitute cobalt ferrite is too high (*x* ≥ 0.4), leading to the B–B interaction increases; consequently, the A–B interaction decreases and subsequent decreases in magnetization.

It is observed from Table 5 that the coercivity of CoFe_2_O_4_ is 1005.32 Oe, and the coercivity is less than 100 Oe with Zn content *x* = 0.5 or 0.6. When *x* = 0.7, the coercivity is nearly zero, indicating the sample has turned from hard to soft magnetic materials. The magnetic state is from ferrimagnetic to superparamagnetic, which is in a good agreement with the result of the Mössbauer spectra. The relationship of the coercivity *H_C_*, the magnetic anisotropy *K*_1_, and the magnetization *M_S_* is [13]:

(7) $$H_{C}=\frac{2K_{1}}{M_{S}}$$

From Table 5, we know that regardless of whether the magnetization decreases or increases, the coercivity decreases by increasing the Zn concentration. As a result, the decrease of coercivity is attributed to the reduction of the magnetic anisotropy. The anisotropy contribution comes from Co^2+^ ions of the octahedral B site, due to the electron configuration of Co^2+^ being 3d^7^ [13], as well as the ion’s spin and incompletely frozen orbital angular momentum coupling [30,31]. The Zn^2+^ of the 3d^10^ electron configuration has a zero angular momentum (l = 0), and it does not contribute to magneto-crystalline anisotropy. From our earlier research [32], the average particle size of CoFe_2_O_4_ (*x* = 0) is between 85.3 and 102.4 nm, and with increasing Zn content the particle size increases [23,33,34]. The sample’s magneto-crystalline anisotropy decreases with increasing Zn^2+^ ions substituted for Co^2+^ ions, with subsequent decreases in the coercivity [35,36]. Kamali et al. synthesized NiFe_2-x_Al_x_O_4_ ferrites and observed the complicated cation distributions in this ferrite system as a function of *x*. The relationship between the electronic ground state, magnetism, and cation distributions is explained in terms of a model [37,38,39].

## 4. Conclusions

The XRD indicates that the ferrite Co_1−*x*_Zn*_x_*Fe_2_O_4_ calcined at 800 °C is a single-phase cubic spinel structure. The increase of the lattice parameter is attributed to replacement of smaller Co^2+^ ions by larger Zn^2+^ ions. The XRD patterns of ferrite Co_0.9_Zn_0.1_Fe_2_O_4_ sintered at different temperature indicate that the ferrite prepared with the experimental method of sol-gel auto-combustion have good crystallinity. Room temperature Mössbauer spectra reveal a transition from ferrimagnetic behavior to superparamagnetic behavior by increasing the zinc concentration for Co_1−*x*_Zn*_x_*Fe_2_O_4_ calcined at 800 °C. The spectra of Co_0.9_Zn_0.1_Fe_2_O_4_ calcined at different temperatures are fitted with two sextet sub-patterns. The variation of the magnetic hyperfine field can be attributed to the average crystallite size change with annealing temperature. The magnetization increases initially (up to *x* = 0.3) and then decreases with increasing Zn content. When *x* = 0.3, the magnetization maximum value is 83.51 emu/g. The change of the magnetization can be explained with Néel’s theory and the Yafet–Kittel model. As Zn content *x* increases, the coercivity and remanence is close to zero, indicating that the sample shows superparamagnetic character.

## Figures and Tables

**Table 1:** XRD date of Co_1−*x*_Zn*_x_*Fe_2_O_4_ calcined at 800 °C.

Content (*x*)	Lattice Parameter (Å)	Average Crystallite Size (Å)	Density (g/cm^3^)
0	8.35497	556	5.3468
0.1	8.39435	508	5.2693
0.2	8.40884	498	5.2421
0.3	8.41082	455	5.2384
0.4	8.41256	489	5.2351
0.5	8.44421	408	5.3187
0.6	8.42766	482	5.3501
0.7	8.45158	360	5.3048

**Table 2:** XRD date of Co_0.9_Zn_0.1_Fe_2_O_4_ sintered at different temperatures.

Temperature (°C)	Lattice Parameter (Å)	Average Crystallite Size (Å)	Density (g/cm^3^)
unsintered	8.38224	270	5.2921
200	8.40569	312	5.2480
400	8.38644	314	5.2842
600	8.38464	332	5.2876
800	8.39435	508	5.2693

**Table 3:** Mössbauer parameters of Co_1−*x*_Zn*_x_*Fe_2_O_4_ sintered at 800 °C.

Content (*x*)	Component	I.S. (mm/s)	Q.S. (mm/s)	H (T)	Γ (mm/s)	A_0_ (mm/s)
0	Sextet (A)	0.237	−0.004	48.946	0.360	33
	Sextet (B)	0.375	−0.024	45.695	0.322	67
0.1	Sextet (A)	0.247	0.019	48.459	0.344	26
	Sextet (B)	0.343	−0.027	45.227	0.378	74
0.2	Sextet (A)	0.248	0.038	47.735	0.386	21
	Sextet (B)	0.340	−0.016	42.532	0.352	79
0.3	Sextet (A)	0.235	0.055	47.508	0.429	11
	Sextet (B)	0.306	−0.050	38.946	0.338	89
0.4	Sextet (B)	0.288	−0.022	34.911	0.375	100
0.5	Sextet (B)	0.309	0.0002	28.6	0.375	100
0.6	Sextet (B)	0.346	−0.005	18.8	0.291	100
0.7	Double	0.347	0.4305	0	0.357	100

**Table 4:** Mössbauer parameters of Co_0.9_Zn_0.1_Fe_2_O_4_ calcined at different temperatures.

Temperature (°C)	Component	I.S. (mm/s)	Q.S. (mm/s)	H (T)	Γ (mm/s)	A_0_ (mm/s)
unsintered	Sextet (A)	0.225	0.056	48.356	0.394	22.5
	Sextet (B)	0.328	−0.050	43.905	0.336	77.5
200	Sextet (A)	0.249	0.007	48.449	0.391	22.5
	Sextet (B)	0.348	−0.011	43.712	0.351	77.5
400	Sextet (A)	0.237	0.016	48.458	0.406	18.9
	Sextet (B)	0.324	−0.030	44.154	0.387	81.1
600	Sextet (A)	0.234	0.008	48.582	0.396	23.1
	Sextet (B)	0.346	0.001	44.563	0.366	76.9

**Table 5:** Magnetic data for Co_1−*x*_Zn*_x_*Fe_2_O_4_ calcined at 800 °C.

Content (*x*)	*M_S_* (emu/g)	*H_C_* (Oe)	*M_r_*^1^ (emu/g)	*n_B_*
0	72.58	1005.33	34.71	3.05
0.1	80.46	703.91	33.14	3.39
0.2	82.61	402.24	26.46	3.49
0.3	83.51	301.75	25.00	3.54
0.4	76.97	100.73	23.64	3.27
0.5	63.63	75.62	15.08	2.71
0.6	41.23	25.39	4.06	1.76
0.7	20.09	0.24	0.13	0.86

^1^ M_r_ is remanent magnetization.
